# Identification of New Natural DNA G-Quadruplex Binders Selected by a Structure-Based Virtual Screening Approach

**DOI:** 10.3390/molecules181012051

**Published:** 2013-09-30

**Authors:** Anna Artese, Giosuè Costa, Francesco Ortuso, Lucia Parrotta, Stefano Alcaro

**Affiliations:** Dipartimento di Scienze della Salute, Università degli Studi “Magna Græcia”, Campus “S. Venuta”, Viale Europa, Germaneto, Catanzaro 88100, Italy

**Keywords:** DNA, G-quadruplex, PDB, pharmacophore, natural compounds, virtual screening, docking

## Abstract

The G-quadruplex DNA structures are mainly present at the terminal portion of telomeres and can be stabilized by ligands able to recognize them in a specific manner. The recognition process is usually related to the inhibition of the enzyme telomerase indirectly involved and over-expressed in a high percentage of human tumors. There are several ligands, characterized by different chemical structures, already reported in the literature for their ability to bind and stabilize the G-quadruplex structures. Using the structural and biological information available on these structures; we performed a high throughput *in silico* screening of commercially natural compounds databases by means of a structure-based approach followed by docking experiments against the human telomeric sequence *d*[AG_3_(T_2_AG_3_)_3_]. We identified 12 best hits characterized by different chemical scaffolds and conformational and physicochemical properties. All of them were associated to an improved theoretical binding affinity with respect to that of known selective G-binders. Among these hits there is a chalcone derivative; structurally very similar to the polyphenol butein; known to remarkably inhibit the telomerase activity.

## 1. Introduction

Telomeres are specialized DNA sequences that cap the ends of linear chromosomes and consist, in human and vertebrate, of highly conserved tandem repeats of the hexanucleotide *d*(TTAGGG)_n_ [1,2,3,4]. The guanines are over-represented not only in telomeres, but also in promoter regions of genes, especially proto-oncogenes, such as c-myc, c-kit, bcl-2, VEGF, H-ras and N-ras, as well as in other human genes [5,6]. The G-rich telomeric sequence can assume G-quadruplex DNA secondary structures (G4s), that consist of stacked G-tetrad planes stabilized by monovalent cations, such as Na^+^ and K^+^ and connected by a network of Hoogsteen’s like hydrogen bonds [7]. G4 structures adopt several topologies and, based on the orientation of the DNA strands, can be classified into various groups. In fact G4 structures can assume parallel, antiparallel or hybrid folds. They can form within one strand (intramolecular) or from multiple strands (intermolecular), and different loop structures are possible [8,9]. In particular the least polymorphic tetramolecular G-quadruplexes adopt only a parallel conformation with all four strands in the same direction [10,11].

The dimerization of two guanine-rich DNA or RNA sequences folded into double-stranded helical duplex structures is responsible for the formation of bimolecular G-quadruplexes in three possible conformations, one parallel and two antiparallel structures. The most polymorphic G-quadruplexes are the unimolecular ones. They are formed from the folding of a single guanine rich DNA or RNA sequence into a four-stranded quadruple helix structure with three connecting loops, that can adopt multiple topologies. G4 structures can be extremely stable, although the topology and stability of the G4 structure depend on many factors, including the length and sequence composition of the total G4 motif, the size and the type of the loops between the guanines (*i.e.*, lateral, diagonal, or double chain-reversal), strand stoichiometry and alignment [12,13,14], glycosyl torsion angles (*i.e.*, *syn* or *anti*) and the nature of the binding cations [15].

The activity of telomerase has been shown to be inhibited by the formation of DNA G-quadruplexes in the human telomere [16,17,18,19]. The enzyme telomerase is an obligate ribonucleoprotein (RNP) whose catalytic function depends minimally on two components: the telomerase reverse transcriptase (TERT) protein and telomerase RNA (known as TR, TER, or TERC). Telomerase is absent for the normal function of most somatic cells that usually have longer telomeres, whereas it is widely expressed in immortal cells [20]. In fact, telomeres are stably maintained in length in 80%–85% of human tumor cells, which divide indefinitely by the action of telomerase [21]. Therefore, since telomerase is necessary for the immortality of many cancer types, it is thought to be a potentially highly selective and attractive drug target for several anti-tumor strategies. Its action is detected in most primary human tumor specimens and tumor-derived cell lines, such as those of the prostate, breast, colon, lung and liver [22]. Hence, inactivation of enzyme may play an important role in cancer therapy.

In cancer treatment the telomerase can be targeted by means of two different strategies. One is based on the direct telomerase inhibition through the block of its catalytic subunit (hTERT) or its RNA template (hTER) and the consequent telomere shortening.

The other one is an indirect approach focused on G-quadruplex stabilizers that, by blocking telomerase access to telomeres or by inhibiting the binding of telomerase-associated proteins, lead to telomere uncapping [23,24].

Some G-quadruplex-interactive compounds have become prospective anticancer agents that display relatively low cytotoxicity [25].

Generally, the development of small molecules as G-quadruplex binders has been largely based on polycyclic planar aromatic compounds with at least one substituent terminating in a cationic group [26,27]. Normally two such substituents are required. The rationale for the planar moiety has been that this would stack effectively onto planar G-quartets, which has been confirmed by several crystallographic and NMR studies of G-quadruplex-ligand complexes [28,29,30,31,32,33]. Several classes of telomerase inhibitors developed to directly inhibit the enzyme are reported in the literature. These compounds are chemically diverse such as porphyrins, perylene diimides, fluoroquinolones, indoloquinolines, cryptolepines, quindolines, phenanthrolines, triazines, carbazole derivatives, ethidium derivatives, bisamido-anthraquinones, fluorenones, acridones and acridines [18]. Nevertheless *in vivo* activity in xenograft cancer models has been reported to date for few telomeric quadruplex ligands, notably the trisubstituted acridine compound BRACO-19 [34], the polycyclic compound RHSP4 [35,36] and telomestatin [37]. However, none of these molecules has progressed beyond the experimental stage into clinical trial, probably in part because these compounds are insufficiently drug-like. Only quarfloxin, a first-in-class G-quadruplex-interactive drug, reached phase II clinical trials [38].

The quadruplex-binding acridine ligands BRACO-19 and RHPS4, in common with telomestatin, induce rapid replicative senescence in cancer cells and activate the same DNA damage response that follows DNA double-strand breaks. Therapeutically effective quadruplex-binding ligands should have minimal duplex DNA affinity and therefore more generalized toxicity. The structural requirements for selectivity have not yet been fully clarified, but mostly involve steric features incompatible with the dimensions of a double helix.

In particular BRACO-19 was one of the series of the compounds designed by computer modeling to exploit the unique structural features of G-quadruplex DNA [39,40]. A di-substituted acridine molecule that was known to bind with quadruplexes was used as a starting model and different substitutions were carried out at the third position. BRACO-19 has very low cytotoxicity, but much higher G-quadruplex-binding and telomerase-inhibitory activity than its parent compound. Subsequently, BRACO-19 was shown to inhibit the catalytic function of telomerase in human cancer cells and destabilize the telomere capping complex [41]. BRACO-19 also induced extensive end-to-end chromosomal fusions consistent with telomere uncapping. A distinct difference between BRACO-19 and direct hTERT inhibitors that result in gradual telomere loss, is that BRACO-19 produces anti-tumor effects soon after treatment. BRACO-19 induced significant tumor regression within 7–10 days of the initiation of treatment in a DU-145 prostate cancer xenograft [42]. Despite all its favorable characteristics, the major limitations of BRACO-19 are its lack of membrane permeability and small therapeutic window. Moreover the three positive charges on the BRACO-19 molecule are probably a factor in the inability of this compound to penetrate larger tumors in both the UXF1138L and A431 xenograft models [34,43].

Other G-binders cannot be used as drugs due to pharmacokinetic and selectivity limitations, since some of them are not able to discriminate between cancer and normal cells; in other cases, e.g., for several anthraquinone, triazole and fluorenone derivatives, in order to obtain the antiproliferative effect onto different tumor lines, large doses are needed [44,45,46]. Recently a new G-quadruplex binding ligand, namely 1*H*-pyrazole-3-carboxy-4-methyl-5-phenyl-(1*H*-indol-3-ylmethylene)hydrazide, was identified from a database of 100,000 drug-like compounds by *in silico* high-throughput docking and was demonstrated experimentally to be an effective stabilizer of G-quadruplex [47]. Therefore, together with the identification and optimization of synthetic molecules able to selectively stabilize the G-quadruplex conformation, in last years the scientific research is increasingly directing towards the natural world with the aim to avoid the synthetic limitations and to reduce some important adverse effects.

For example fonsecin B has been identified as stabilizing ligand of c-myc G-quadruplex DNA using high-throughput virtual screening of a natural product database, and inhibited Taq polymerase-mediated DNA extension *in vitro* through stabilization of the G-quadruplex secondary structure [48].

Effectively, also our research group carried out several studies focused on the G-quadruplex target by analyzing both selective synthetic and natural G-binders [49,50,51,52,53,54,55]. In a recent publication we identified a new promising scaffold for G-quadruplex characterized by a psoralen moiety performing a high throughput *in silico* screening of commercially available molecules databases by merging ligand- and structure-based approaches by means of docking experiments [56]. Conversely, in this manuscript the available structural and biological information on G-quadruplex structures allowed us to perform a high throughput *in silico* screening of commercially natural compounds databases by means only of a structure-based approach followed by docking experiments against the human telomeric sequence *d*[AG_3_(T_2_AG_3_)_3_]. Twelve best candidates, associated to an improved theoretical binding affinity with respect to that of known selective G-binders, were identified and their biophysical and biological assays are actually in progress.

## 2. Results and Discussion

### 2.1. Pharmacophore Models

For each analyzed complex, we generated several pharmacophore hypotheses based on the structural information reported in literature. In order to validate our models, we used datasets of active, inactive and decoys compounds and we evaluated the ROC curve. Receiver operating characteristic (ROC) curves have been widely used to evaluate VS methods [57,58]. An ROC curve is a plot of true-positive rates *versus* false-positive rates for all compounds. The area under the ROC (AU-ROC) curve is the probability of active compounds being ranked earlier than decoy compounds.

For the 3CE5 [31] PDB [59] model, where BRACO-19 is complexed to a bimolecular human telomeric G-quadruplex of sequence *d*(TAGGGTTAGGGT) in parallel conformation, the first pharmacophore model generated by LigandScout [60] was represented by 10 features (three aromatic interactions, two hydrogen bond acceptors and five hydrogen bond donors). By means of manual modifications, we generated the two optimized pharmacophore hypotheses reported in Table 1.

**Table 1:** Classification of the pharmacophore hypotheses generated by 3CE5 PDB model.

3CE5 PDB model					
Hypothesis	# Features	Active compounds	% Active	Inactive compounds	% Inactive
H_1	4	42	23.3	2	0.14
H_2	3	71	39.4	12	1.17

**Figure 1:**
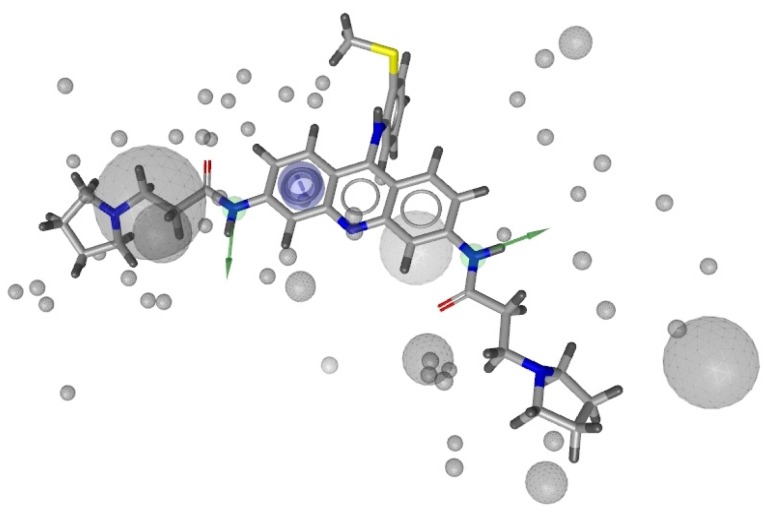
representation of the pharmacophore H_2 generated starting from 3CE5 PDB model by LigandScout software.

After validation, we selected H_2 (Figure 1), composed by one aromatic interaction and two hydrogen bond donors, as the best one.

**Figure 2:**
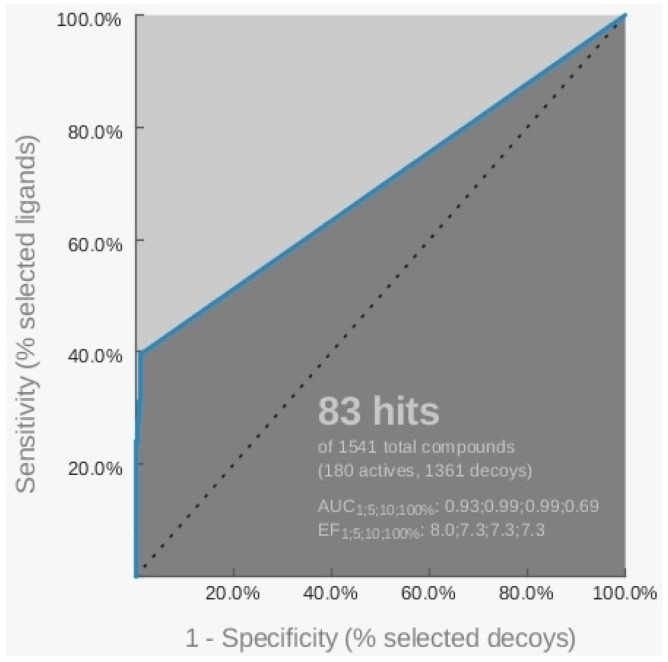
Representation of the ROC curve related to the pharmacophore H_2 generated starting from the 3CE5 PDB model by LigandScout software.

The ROC curve related to this hypothesis indicated a sensitivity equal to 39.4%, as shown in Figure 2, since the model was able to recognize 71 active and 12 inactive compounds.

For the 3SC8 [61] PDB structure, where an intramolecular human telomeric DNA G-quadruplex in parallel conformation is in complex with two tetra-substituted naphthalene diimides (NDI), the first pharmacophore model generated automatically by LigandScout was characterized by nine features (two aromatic interactions, one hydrogen bond acceptor, three hydrogen bond donors, three positively ionizable groups). The complexity of such a model did not allow to find any of the compounds included in the test dataset. Therefore, after appropriate simplifications, we obtained the seven pharmacophore hypotheses described in detail in Table 2.

**Table 2:** Classification of the pharmacophore hypotheses generated by 3SC8 PDB model.

3SC8 PDB model					
Hypothesis	# Features	Active compounds	% Active	Inactive compounds	% Inactive
H_1	3	73	40.5	90	6.61
H_2	4	4	2.22	14	1.03
H_3	3	73	40.5	4	0.29
H_4	3	73	40.5	60	4.41
H_5	3	74	41.1	45	3.31
H_6	3	74	41.1	26	14.4
H_7	3	74	41.1	84	6.17

**Figure 3:**
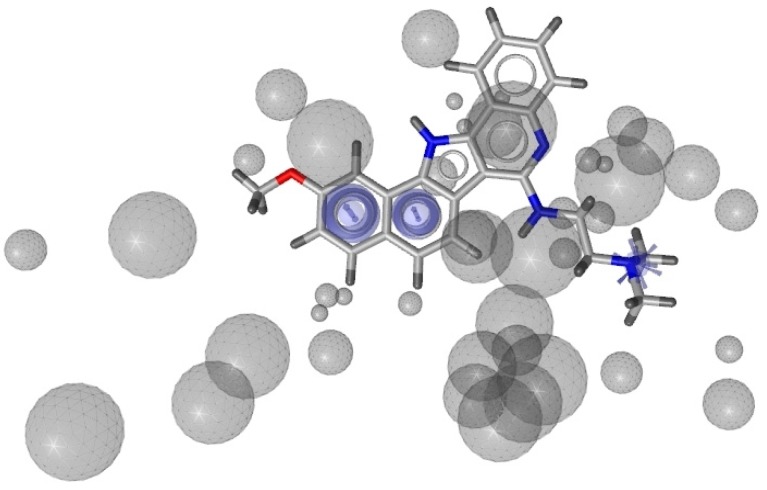
Representation of the pharmacophore H_3 generated starting from 3SC8 PDB model by LigandScout software.

After validation, H_3 (Figure 3), based on two aromatic interactions and one positively ionizable group, was chosen as the most promising.

**Figure 4:**
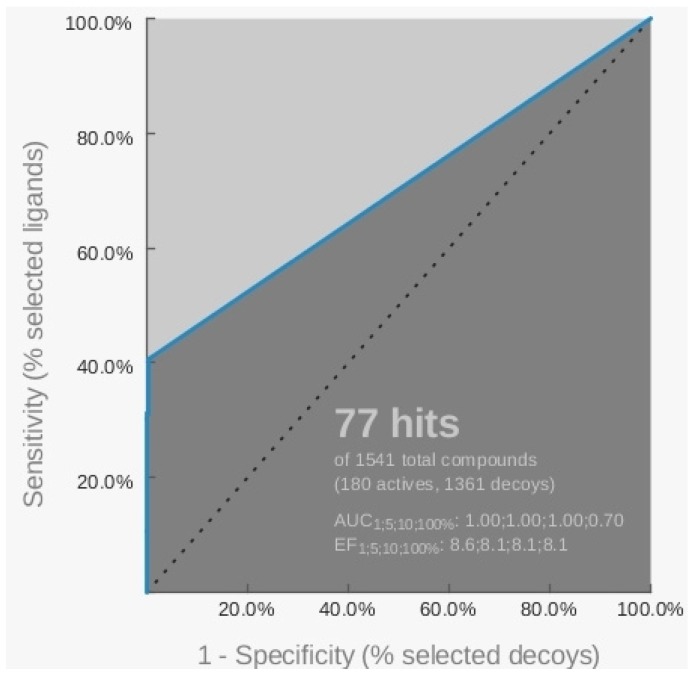
Representation of the ROC curve related to the pharmacophore H_3 generated starting from the 3SC8 PDB model by LigandScout software.

The sensitivity obtained by this hypothesis, able to find 73 active and four inactive compounds, was equal to 40.5%, as indicated by the ROC curve (Figure 4).

As regards the 4FXM [62] PDB structure, that is the X-ray crystal structure of a complex between the N-methyl mesoporphyrin IX (NMM) and human telomeric DNA sequence, the pharmacophore model generated automatically by LigandScout presented five features (three aromatic interactions and two hydrogen bond acceptors); after manual modifications, we achieved nine pharmacophore hypotheses reported in Table 3.

**Table 3:** Classification of the pharmacophore hypotheses generated by 4FXM PDB model.

4FXM PDB model					
Hypothesis	# features	Active compounds	% Active	Inactive compounds	% Inactive
H_1	3	17	9.4	11	0.8
H_2	3	109	60.5	496	36.4
H_3	3	107	59.4	476	35
H_4	3	101	56.1	460	33.8
H_5	3	94	52.2	240	17.6
H_6	3	94	52.2	204	15
H_7	3	99	55	113	8.3
H_8	3	99	55	136	10
H_9	3	90	50	73	5.63

Among them, H_9, shown in Figure 5 and based on two aromatic interactions and an hydrogen bond acceptor, resulted the best one after the validation step.

**Figure 5:**
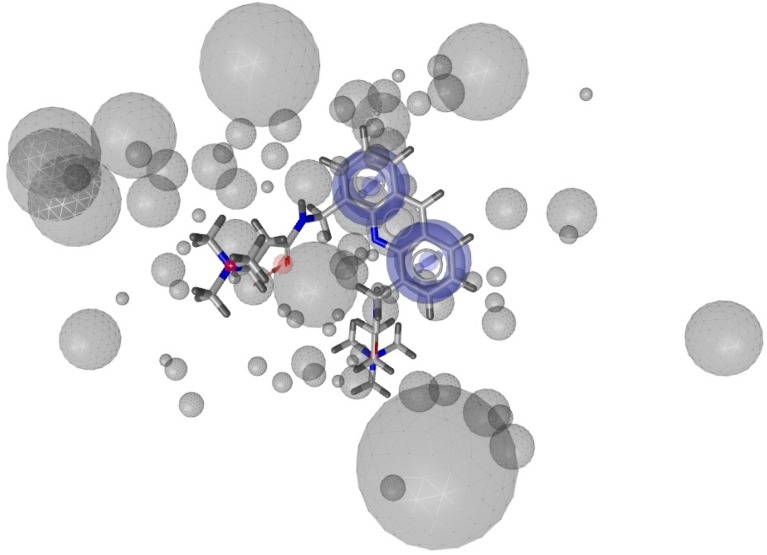
Representation of the pharmacophore H_9 generated starting from the 4FXM PDB model by LigandScout software

The ROC curve related to this hypothesis indicated a sensitivity equal to 50.0%, as shown in Figure 5, since the model was able to recognize 90 active and 73 inactive compounds.

**Figure 5:**
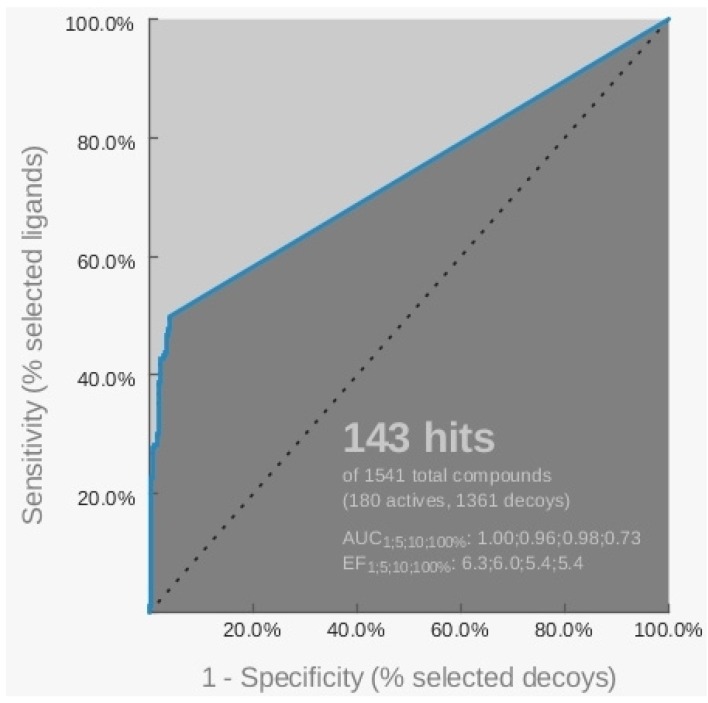
Representation of the ROC curve related to the pharmacophore H_9 generated starting from the 4FXM PDB model by LigandScout software.

### 2.2. Virtual Screening of the Natural Database

The three best pharmacophore hypotheses obtained after the validation procedure were used for virtually screening the library of 31,041 natural compounds. In particular, as reported in Table 4, our search, performed separately for the three adopted models, allowed us to identify a different number of ligands.

**Table 4:** Number of ligands screened by the natural database by means of the single pharmacophore models generated starting from the experimental structures 3CE5, 3SC8 and 4FXM.

Model	Screened ligands
3CE5	673
3SC8	73
4FXM	4013

After the virtual screening procedure, we analyzed the LigandScout fit scores for all the identified ligands, but we observed no significant differences among them. Thus we decided to perform Glide HTVS [63] docking simulations. In order to choose the G-quadruplex model able to better discriminate the binding affinity between actives and decoys, we used the three X-ray experimental structures adopted for the pharmacophore model generation, after removing their complexed ligands. We found 3CE5 as the best receptor and, as it can be noticed in the Figure S1, applying a cut-off of −7.0 kcal/mol, a small number of decoys was selected, while most of actives were included. Subsequently, we evaluated the molecular recognition of our library towards 3CE5 G-quadruplex model, by including also the co-crystallized tri-substituted acridine BRACO-19.

**Figure 6:**
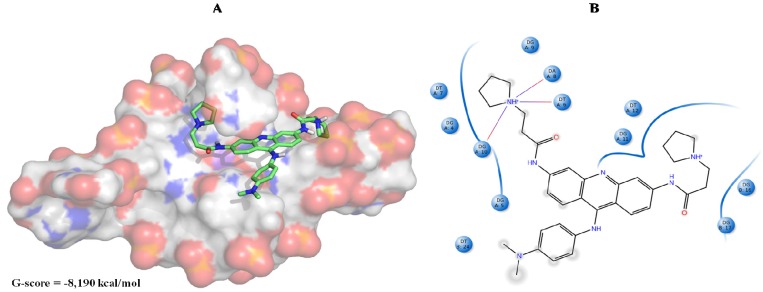
(**A**) BRACO-19 best pose against the 3CE5 PDB model obtained by Glide HTVS docking method. (**B**) BRACO-19 best pose interaction diagram 2D representation. The red lines and the grey spheres indicate, respectively, the salt bridge contacts and the solvent exposure areas.

Within this cut-off value, from the initial 4759 compounds obtained after the virtual screening of the natural database, only 230 were finally selected, and among them 47, seven and 176 were from 3CE5, 3SC8 and 4FXM screening, respectively. In order to further filter them, we considered only those ligands whose binding affinity towards 3CE5 model was better if compared to that of BRACO-19 (Figure 6).

As shown in Figure 6B, BRACO-19 can establish several Van der Waals interactions with different G4 nucleobases and it is mainly stabilized by three salt bridge contacts with adenine, thymine and guanine, respectively, at positions 8, 6 and 10.

The twelve theoretically most promising hits are reported in Table 5 with their G-scores. Among them, two were found starting from the pharmacophore model generated by the 3CE5 structure, while the other 10 ligands were identified using the 4FXM best hypothesis. We highlighted that eight of the 12 hits resulted from both adopted experimental models.

**Table 5:** 2D structure of the best hits identified into the natural database and filtered according to BRACO-19 G-score after their molecular recognition against 3CE5 model. The asterisk labels those compounds screened by means of the pharmacophore model generated by 3CE5 structure. The G-scores are expressed in kcal/mol.

*Best hits*		
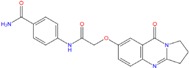	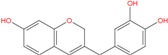	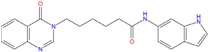
ZINC79190432 −8.451	ZINC77031588 * −8.509	ZINC32124244 −8.485
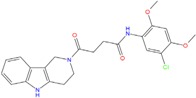	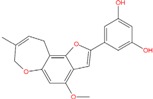	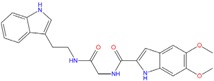
ZINC20760949 −8.429	ZINC14610063 −8.334	ZINC12902036 −9.082
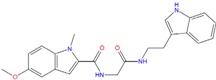	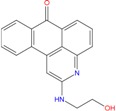	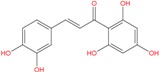
ZINC12664647 −8.378	ZINC12377179 −8.828	ZINC04252698 * −8.206
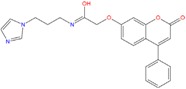	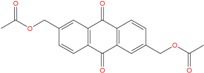	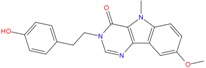
ZINC03985155 −8.609	ZINC03843477 −8.215	ZINC02131213 −8.944

* compounds obtained from the pharmacophore model based on the 3CE5 PDB entry.

**Figure 7:**
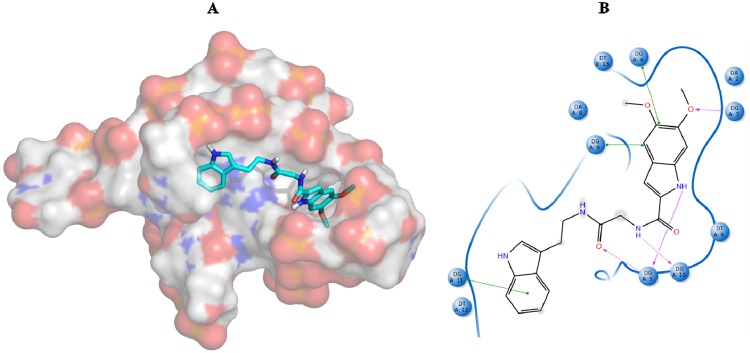
(**A**) ZINC12902036 best pose against 3CE5 PDB model obtained by Glide HTVS docking method. (**B**) ZINC12902036 best pose interaction diagram 2D representation. The green lines, the magenta arrows and the grey spheres indicate, respectively, the π-π stacking interactions, the hydrogen bond contacts and the solvent exposure areas.

Analyzing the best poses of our hits, we observed that all of them were able to better recognize the bottom site [55] of the G-quadruplex conformation, with the bis-indole ZINC12902036 having an improved affinity if compared to BRACO-19 due to additional contacts into the lateral loop (Figure 7).

As reported in Figure 7B, ZINC12902036 hydrophobic moieties are involved in three π-π stacking interactions with guanines, respectively, at positions 4, 9 and 11. Furthermore, the bis-indole ligand is well accommodated into the target by four hydrogen bonds with G3, G5 and G10, responsible for its better affinity with respect to that of the reference tri-substituted acridine.

Finally, with the aim to theoretically evaluate the chemical and physical properties of the 12 best hits, we used the Canvas software [64], that allowed us to calculate their molecular weight, lipophilicity, hydrogen bond acceptor groups, hydrogen bond donor groups and polar surface area (Table S1).

Analyzing the chemical scaffolds of the best hits identified by our virtual screening approach, we observed that ZINC79190432 and ZINC32124244 belong to the quinazoline alkaloids, while ZINC12377179 is characterized by a quinoline moiety. In particular this class of ligands was associated to antiviral and anticancer activities [65].

By contrast, ZINC77031588 and ZINC03985155 are based on a chromene structure, already reported in several G-binders related to the Green tea catechins [66], known as cancer preventive agents.

Moreover, anthraquinone-containing extracts from different plant sources have been found to have wide variety of pharmacological activities such as antiinflammatory, wound healing, analgesic, antipyretic, antimicrobial, and antitumor activities [67]. In particular several anthraquinone derivatives were used as chemotherapeutic agents in anticancer treatments and are potent human telomerase inhibitors [68,69,70]. The identified ZINC03843477 ligand is associated to the anthraquinone chemical scaffold and could be a potential G-binder.

On the other hand, ZINC20760949 is related to a tetrahydro-β-carboline chemical structure, present in several fruits and vegetables [71,72] and widely used in folk medicine in anti-inflammatory therapy. However, recently (3*S*)-1,2,3,4-tetrahydro-β-carboline-3-carboxylic acid, an amino acid isolated from *C. endivia.* L., was found for the first time to show cytotoxic activity in colorectal tumor cell line HCT-8 [73], and thus it can be considered as a potential candidate for developing chemotherapeutic drugs against cancer.

One of the most interesting hit resulted the chalcone ZINC04252698, structurally very similar to the polyphenol butein. This compound, extracted from *T. vernicifluum* medicinal plant and known for its antioxidant properties [74], was found to down-regulate hTERT gene expression, to decrease telomerase activity and also to suppress expression of c-myc at the transcriptional level [75].

Our results focused some chemical scaffolds already known for their selectivity against DNA G-quadruplex conformation. In particular, quinazoline, quinoline, anthraquinone and bis-indole derivatives were tested for their ability to inhibit the telomerase enzyme and for their cytotoxic effect against some tumor cell lines [76]. These observations, together with the recent finding that butein remarkably decreases telomerase activity, allow us to feel confident with further results currently in progress on the twelve compounds by means of biophysical and biological assays.

## 3. Experimental

### 3.1. Dataset of Active, Inactive and Decoys Compounds

The active compounds used in this study were obtained from the ChEMBL database [77]. We firstly indicated the telomerase as target and then selected 1,350 molecules from various literature sources. In order to filter them, we chose those molecules whose IC_50_ values were lower than 10 μM, obtaining an active test set of 180 compounds. For the selection of the inactive molecules, we applied the same approach, thus filtering 37 compounds characterized by IC_50_ values higher than 10 μM. Our choice was based on the same experimental assay and conditions. In Table S2 the chEMBL code, the SMILES chemical formula [78,79] and the IC_50_ value of the active compounds are reported.

Unfortunately, we have not any decoys set for the telomerase target. However, we used a focused library already reported in our recent publication [56] that reflected the physicochemical properties of the active set used in that analysis [80]. In particular, we had considered Pubchem representative compounds at 90% Tc (~150,000 compounds), and filtered them applying the same protocol adopted for the other databases [81]. One thousand three hundred and twenty four compounds were selected and considered as “decoys” set.

All the molecules used as test sets were submitted to 2000 iterations of full energy minimization adopting the Polake-Ribiere Conjugated Gradient (PRCG) algorithm and the “all atoms” notation of the MMFF force field [82]. Solvent effects were considered by adopting the implicit solvation model GB/SA water [83]. The optimization process was performed up to the derivative convergence criterion of 0.05 kcal Å^−1^·mol^−1^.

### 3.2. Database of Natural Compounds

The high throughput *in silico* approach was conducted by virtually screening ~210,000 natural compounds publicly available in the ZINC [81] repository associated with nine vendor companies (Ambinter Natural Products [84], AnalytiCon Discovery NP [85], IBScreen NP [86], Indofine Natural Products [87], Molecular Diversity Preservation International [88], Nubbe Natural Products [89], Princeton NP [90], Selleck BioChemicals NP [91], Specs Natural Products [92], TCM Database@Taiwan [93] and UEFS Natural Products [94]). Instant JChem v. 5.12.3.1 [95] allowed us to deduplicate the included compounds, thus obtaining 198,306 molecules. They were subsequently filtered by drug-like properties and only those with no chiral centers were chosen. Starting from the 31,041 final molecules, a conformers library was generated by LigandScout OMEGA-best software [96,97] using the default parameters.

### 3.3. Pharmacophore Models Generation and Virtual Screening

For building the pharmacophore models used in this study and for performing the virtual screening we adopted LigandScout v. 3.1 [60], a software tool that allows to rapidly and transparently derive 3D chemical feature-based pharmacophores from structural data of macromolecule/ligand complexes in a fully automated and convenient way. In our analysis, we started from three PDB [59] experimental G-quadruplex complexes and we generated several pharmacophore hypotheses by a structure-based approach. In particular, we adopted the X-ray structures with the following PDB ID: 3SC8 [61], 4FXM [62], 3CE5 [31]. 3SC8 is an intramolecular human telomeric DNA G-quadruplex in parallel conformation in complex with two tetra-substituted naphthalene diimides (NDI) functionalized with positively charged *N*-methyl-piperazine side-chains. The NDI molecules in both structures are stacked effectively over the terminal 3' G-quartet surfaces, with extensive π-π contacts, resulting in a 1:1 quadruplex:ligand stoichiometry. There are significant differences between the complexes in terms of ligand mobility and interactions within G-quadruplex grooves. One of the two ligands is markedly less mobile in the crystal complex and is more G-quadruplex-stabilizing, forming multiple electrostatic/hydrogen bond contacts with quadruplex phosphate groups [61].

4FXM is the X-ray crystal structure of a complex between the N-methyl mesoporphyrin IX (NMM) and human telomeric DNA sequence. NMM is able to adjust its macrocycle geometry to closely match that of the terminal G-tetrad required for efficient π-π stacking. The out-of-plane N-methyl group of NMM fits perfectly into the center of the parallel guanine core, where it aligns with potassium ions and the molecule results well stabilized by means of hydrophobic contacts. In contrast, the interaction of the N-methyl group with duplex DNA or antiparallel G-quadruplex would lead to steric clashes that prevent NMM from binding to these structures, thus explaining its unique selectivity towards the parallel conformation [62].

3CE5 is a complex between a bimolecular human telomeric G-quadruplex of sequence *d*(TAGGGTTAGGGT) in parallel conformation and BRACO-19. Each bimolecular quadruplex in this structure contains three planar stacked G-tetrads with a BRACO-19 molecule stacking directly onto the 3' end G-tetrad face. The drug is asymmetrically stacked on the G-tetrad at the end of one quadruplex, showing π-π overlap with just two guanine bases and the cationic ring nitrogen atom of the acridine ring in-line with the K^+^ ion channel that runs through the quadruplex [31].

During the initial validation step, the first generated pharmacophore models, obtained from each of the three PDB complexes, were associated to poor performance. Therefore, we manually modified them by including or removing some features, by increasing or reducing the tolerance and by adjusting the exclusion volume spheres, with the aim to better characterize the binding site. In this way, we obtained optimized pharmacophore hypotheses, subsequently evaluated by their Receiver Operating Characteristic (ROC) curve profile, and used for the virtual screening of the natural database.

### 3.4. Docking Experiments

All docking and scoring calculations were performed using Glide version 5.8 HTVS mode [63]. DNA models were submitted to map calculations using a box of about 64,000 Å^3^, centered onto the centroid of the receptor. All default settings were used for docking.

The filtered 31,041 ligands were docked flexibly, generating 10 poses per ligand. Since full force-field minimization was not performed during the docking stage, a post-docking minimization was applied. In this energy minimization the ligand geometries were optimized and re-scored.

The binding pocket characteristics and the protein-ligand interactions were identified with automatically-generated Maestro [98] 2D ligand interaction diagrams. These 2D representations of the binding pocket use distinctive colors and shapes to convey binding pocket shape, electrostatics and protein-ligand interactions.

## 4. Conclusions

In this manuscript we developed some pharmacophore models starting from three experimental DNA G-quadruplex complexes. The best hypotheses, validated by the ROC analysis, were used for the virtual screening of natural compounds database extracted from ZINC. A first cutoff value was developed using a large set of active/inactive compounds and adopted for the selection of the screened ligands. A second filtering criterion was based on the re-docked BRACO-19 reference compound score and applied to reduce the first identified molecules. Finally, twelve compounds satisfied all criteria and, interestingly, most of them contain chemical scaffolds already associated with G-quadruplex binding properties and antiproliferative effects.

These hits are currently under experimental investigations in order to complete their biophysical and biological profiles. The most promising ligands will be object of further chemical optimization against the G-quadruplex targets.

## Supplementary Materials

Supplementary materials can be accessed at: http://www.mdpi.com/1420-3049/18/10/12051/s1.
