# Supplementary material for: Identification of New Natural DNA G-Quadruplex Binders Selected by a Structure-Based Virtual Screening Approach
Source: Molecules. 2013 Sep 30;18(10):12051–70. doi: 10.3390/molecules181012051 (PMC6270229; doi:10.3390/molecules181012051)
Supplement: Supplementary file 1 [file molecules-18-12051-s001.pdf]

Supplementary Materials

Figure S1. Docking scores of actives and decoys.

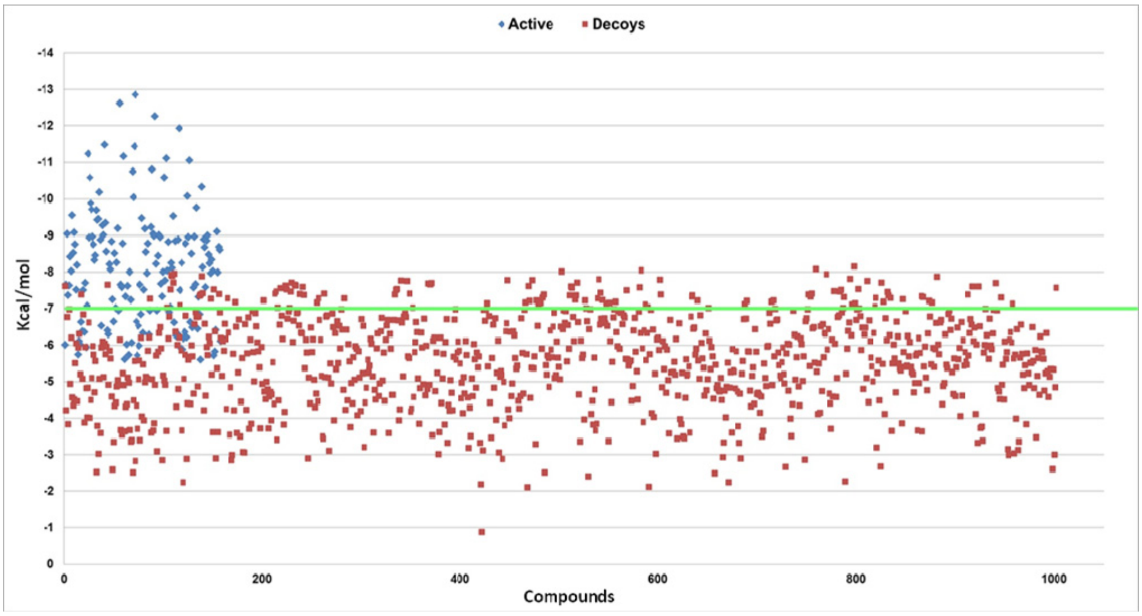

Table S1. Chemical and physical properties of the 12 best hits. MW, LogP, HBA, HBD and PSA indicate, respectively, molecular weight, lipophilicity, hydrogen bond acceptor groups, hydrogen bond donor groups and polar surface area.

Hit	MW	LogP	HBA	HBD	PSA
ZINC79190432	378.38	1.29	5	2	116.31
ZINC77031588	270.28	3.17	4	3	69.92
ZINC32124244	374.44	3.48	3	2	79.78
ZINC20760949	441.91	2.79	4	2	83.66
ZINC14610063	338.35	4.41	5	2	72.06
ZINC12902036	420.46	1.95	4	4	108.24
ZINC12664647	404.46	2.46	3	3	88.15
ZINC12377179	290.32	2.92	3	2	62.22
ZINC04252698	288.25	2.26	6	5	118.22
ZINC03985155	403.43	2.51	6	1	89.85
ZINC03843477	352.34	2.36	6	0	86.74
ZINC02131213	349.38	3.9	4	1	69.28

Table S2. SMILES chemical formula, reference code and IC_{50} values of the active compounds.

**SMILES chemical formula**	**Reference code and IC_{50}**
c1cccc2c1c(c3n2[c@]45c)c6c(c(=o)nc6)c7c8c(cccc8)n(c37)[c@h](o5)c[c@h](nc)[c@@h]4oc	**CHEMBL162 [1]** IC_{50} = 8,32 μM
cn(c)ccnc1nc(cccc2)c2c3[nh]c(c4c13)c5c(cc4)ccc(c5)oc	**CHEMBL10763 [2]** IC_{50} = 9,8 μM
c1cccc[n+]1(c)ccc(=o)nc(c2)ccc(c23)c(=o)c4c(c3=o)cc(cc4)nc(=o)cc[n+]5(c)ccccc5	**CHEMBL14832 [3]** IC_{50} = 7,8 μM
c1cccc(c12)ccc(c2)c(\c)=c\c(=o)nc(c3c(=o)o)cccc3	**CHEMBL27323 [4]** IC_{50} = 5 μM
c1ccccn1ccc(=o)nc(c2)ccc(c23)c(=o)c4c(c3=o)ccc(c4)nc(=o)ccn5ccccc5	**CHEMBL33618 [3]** IC_{50} = 4,5 μM
cc[n+]1ccccc1/c=c2/n(cc)c(=o)/c(s2)=c(/n3c)sc(c34)cccc4	**CHEMBL33859 [3]** IC_{50} = 5 μM
ccn(cc)ccc(=o)nc(c1)ccc(c12)c(=o)c3c(c2=o)ccc(c3)nc(=o)ccn(cc)cc	**CHEMBL34683 [3]** IC_{50} = 3,5 μM
o=p(o)(o)o[p@](=o)(o)o[p@](=o)(o)oc[c@h]1cc[c@h](o1)n(cn2)c(c23)nc(n)[nh]c3=o	**CHEMBL54224 [5]** IC_{50} = 8,6 μM
c1cccn1ccc(=o)nc(cc2)cc(c23)nc4c(c3)ccc(c4)nc(=o)ccn5cccc5	**CHEMBL79900 [6]** IC_{50} = 5,2 μM
c1ccccn1ccc(=o)nc(cc2)cc(c23)nc4c(c3)ccc(c4)nc(=o)ccn5ccccc5	**CHEMBL81268 [7]** IC_{50} = 2,8 μM
oc[c@h]1ccccn1ccc(=o)nc(cc2)cc(c23)nc4c(c3)ccc(c4)nc(=o)ccn5cccc[c@@h]5co	**CHEMBL81271 [7]** IC_{50} = 5,4 μM
oc1ccn(cc1)ccc(=o)nc(cc2)cc(c23)nc4c(c3)ccc(c4)nc(=o)ccn(cc5)ccc5o	**CHEMBL81516 [8]** IC_{50} = 8 μM
cc[c@h]1ccccn1ccc(=o)nc(cc2)cc(c23)nc4c(c3)ccc(c4)nc(=o)ccn5cccc[c@@h]5cc	**CHEMBL81747 [7]** IC_{50} = 2,7 μM

Table S2. *Cont.*

SMILES chemical formula	Reference code and IC_{50}
c1ccccn1ccc(=o)nc(cc2)cc(c23)nc4c(c3)ccc(c4)nc(=o)ccn5cccccc5	**CHEMBL82008 [8]** IC_{50} = 3,1 μM
c[c@@h]1cccn(c1)ccc(=o)nc(cc2)cc(c23)nc4c(c3)ccc(c4)nc(=o)ccn(c5)ccc[c@h]5c	**CHEMBL83173 [8]** IC_{50} = 1,35 μM
c1cccc(c12)n(cc2c)cccn(c(c34)cccc4)cc3cc(=o)ncccccccccccco[p@](=o)(o)oc5c(cl)cccc5	**CHEMBL83388 [9]** IC_{50} = 3,6 μM
cc(c)(c)oc(=o)n1ccc[c@h]1c(=o)n[c@@h](cc2c[nh]c(c23)cccc3)c(=o)ncccccccccccco[p@@](=o)(o)oc4c(cl)cccc4	**CHEMBL86984 [9]** IC_{50} = 9,5 μM
c1ccccc1coc(=o)cc[c@@h](nc(=o)oc(c)(c)c)c(=o)n[c@@h](cc2c[nh]c(c23)cccc3)c(=o)ncccccccccccco[p@](=o)(o)oc4c(cl)cccc4	**CHEMBL87237 [9]** IC_{50} = 6,4 μM
c1ccccn1ccc(=o)nc(c2)ccc(c23)c(=o)c4c(c3=o)cc(cc4)nc(=o)ccn5ccccc5	**CHEMBL89250[10]** IC_{50} = 3,1 μM
cn(c)ccc(=o)nc(c1)ccc(c12)c(=o)c3c(c2=o)cc(cc3)nc(=o)ccn(c)c	**CHEMBL89977 [9]** IC_{50} = 4,7 μM
c1cccc[n+]1(c)ccc(=o)nc(c2)ccc(c23)c(=o)c4c(c3=o)cc(cc4)nc(=o)cc[n+]5(c)ccccc5	**CHEMBL90901 [10]** IC_{50} = 7,8 μM
ccn(cc)ccc(=o)nc(c1)ccc(c12)c(=o)c3c(c2=o)cc(cc3)nc(=o)ccn(cc)cc	**CHEMBL91163 [3]** IC_{50} = 4,3 μM
c1ccccn1ccc(=o)nc(cc2)cc(c2-3)c(=o)c4c3ccc(c4)nc(=o)ccn5ccccc5	**CHEMBL91935 [10]** IC_{50} = 9 μM
ccoc(c1)ccc(c12)nc3c(c2n)ccc(c3)n	**CHEMBL94007 [11]** IC_{50} = 8,2 μM
c1ccccn1ccc(=o)nc(c2)ccc(c23)c(=o)c4c(c3=o)ccc(c4)nc(=o)ccn5ccccc5	**CHEMBL109382 [5]** IC_{50} = 4,5 μM
c[n+](c)(c)ccc(=o)nc1ccc(nc(=o)cc[n+](c)(c)c)c(c12)c(=o)c3c(c2=o)cccc3	**CHEMBL111417 [8]** IC_{50} = 7 μM
c1cccn1ccc(=o)nc(cc2)cc(c23)nc4c(c3nc5c(sc)cccc5)ccc(c4)nc(=o)ccn6cccc6	**CHEMBL137809 [7]** IC_{50} = 0,15 μM

Table S2. *Cont.*

**SMILES chemical formula**	**Reference code and IC_{50}**
c1cccn1ccc(=o)nc(c2)ccc(c23)nc4c(c3nccn(c)c)cc(cc4)nc(=o)ccn5cccc5	**CHEMBL137928 [7]** IC_{50} = 0,57 μM
c1cccn1ccc(=o)nc(c2)ccc(c23)nc4c(c3nc5ccccc5)ccc(c4)nc(=o)ccn6cccc6	**CHEMBL137973 [7]** IC_{50} = 0,21 μM
c1cccn1ccc(=o)nc(c2)ccc(c23)nc4c(c3ncccn(c)c)ccc(c4)nc(=o)ccn5cccc5	**CHEMBL138187 [7]** IC_{50} = 0,08 μM
c1cccn1ccc(=o)nc(c2)ccc(c23)nc4c(c3nc(cc5)ccc5n(c)c)ccc(c4)nc(=o)ccn6cccc6	**CHEMBL138357 [7]** IC_{50} = 0,17 μM
c1cccn1ccc(=o)nc(c2)ccc(c23)nc4c(c3nc5ccccc5)cc(cc4)nc(=o)ccn6cccc6	**CHEMBL138369 [7]** IC_{50} = 1,29 μM
c1cccn1ccc(=o)nc(cc2)cc(c23)nc4c(c3nc(ccc5)cc5n(c)c)ccc(c4)nc(=o)ccn6cccc6	**CHEMBL138487 [7]** IC_{50} = 0,1 μM
c1cccn1ccc(=o)nc(cc2)cc(c23)nc4c(c3nc5cc(sc)ccc5)ccc(c4)nc(=o)ccn6cccc6	**CHEMBL138761 [7]** IC_{50} = 0,1 μM
c1cccn1ccc(=o)nc(cc2)cc(c23)nc4c(c3nc5cc5)ccc(c4)nc(=o)ccn6cccc6	**CHEMBL138811 [7]** IC_{50} = 0,05 μM
c1cccn1ccc(=o)nc(cc2)cc(c23)nc4c(c3nc5ccccc5)ccc(c4)nc(=o)ccn6cccc6	**CHEMBL139287 [7]** IC_{50} = 0,09 μM
c1cccn1ccc(=o)nc(c2)ccc(c23)nc4c(c3nc(ccc5)cc5n(c)c)cc(cc4)nc(=o)ccn6cccc6	**CHEMBL139443 [7]** IC_{50} = 0,6 μM
c1cccn1ccc(=o)nc(c2)ccc(c23)nc4c(c3nc5cc(n)ccc5)cc(cc4)nc(=o)ccn6cccc6	**CHEMBL139500 [7]** IC_{50} = 1,09 μM
c1cccn1ccc(=o)nc(cc2)cc(c23)nc4c(c3nc5c(n)cccc5)ccc(c4)nc(=o)ccn6cccc6	**CHEMBL139511 [7]** IC_{50} = 0,02 μM
c1cccn1ccc(=o)nc(c2)ccc(c23)nc4c(c3nccn(c)c)ccc(c4)nc(=o)ccn5cccc5	**CHEMBL140084 [7]** IC_{50} =0,27 μM
c1cccn1ccc(=o)nc(cc2)cc(c23)nc4c(c3nccn(c)c)ccc(c4)nc(=o)ccn5cccc5	**CHEMBL140180 [7]** IC_{50} = 0,01 μM

Table S2. *Cont.*

**SMILES chemical formula**	**Reference code and IC_{50}**
c1cccn1ccc(=o)nc(c2)ccc(c23)nc4c(c3nc5ccccc5)ccc(c4)nc(=o)ccn6cccc6	**CHEMBL140354 [7]** IC_{50} = 1,33 μM
c1cccn1ccc(=o)nc(cc2)cc(c23)nc4c(c3nccn5ccccc5)ccc(c4)nc(=o)ccn6cccc6	**CHEMBL141540 [7]** IC_{50} = 0,05 μM
c1cccn1ccc(=o)nc(cc2)cc(c23)nc4c(c3nc(ccc5)cc5nc(=o)c)ccc(c4)nc(=o)ccn6cccc6	**CHEMBL141661 [7]** IC_{50} = 0,1 μM
c1cccn1ccc(=o)nc(c2)ccc(c23)nc4c(c3nc5ccc(cc5)oc)cc(cc4)nc(=o)ccn6cccc6	**CHEMBL141740 [7]** IC_{50} = 0,46 μM
c1cccn1ccc(=o)nc(c2)ccc(c23)nc4c(c3nc5ccc(n)cc5)cc(cc4)nc(=o)ccn6cccc6	**CHEMBL142036 [7]** IC_{50} = 0,2 μM
c1ccc[n+]1(c)ccc(=o)nc2ccc(c(c23)c(=o)c4c(c3=o)cccc4)nc(=o)cc[n+]5(c)cccc5	**CHEMBL143452 [3]** IC_{50} = 5 μM
c1ccc[n+]1(c)ccc(=o)nc2cccc(c23)c(=o)c4c(c3=o)c(ccc4)nc(=o)cc[n+]5(c)cccc5	**CHEMBL144219 [3]** IC_{50} = 8,2 μM
cc[n+](c)(cc)ccc(=o)nc1ccc(nc(=o)cc[n+](c)(cc)cc)c(c12)c(=o)c3c(c2=o)cccc3	**CHEMBL144303 [8]** IC_{50} = 3,1 μM
c1cccc[n+]1(c)ccc(=o)nc2cccc(c23)c(=o)c4c(c3=o)cccc4nc(=o)cc[n+]5(c)ccccc5	**CHEMBL144334 [8]** IC_{50} = 8,6 μM
c1ccccn1ccc(=o)nc2cccc(c23)c(=o)c4c(c3=o)c(ccc4)nc(=o)ccn5ccccc5	**CHEMBL144386 [8]** IC_{50} = 3,7 μM
ccn(cc)ccc(=o)nc1cccc(c12)c(=o)c3c(c2=o)cccc3nc(=o)ccn(cc)cc	**CHEMBL144664 [8]** IC_{50} = 2,7 μM
c1cccn1ccc(=o)nc(c2)ccc(c23)c(=o)c4c(c3=o)cc(cc4)nc(=o)ccn5cccc5	**CHEMBL144757 [12]** IC_{50} = 6,7 μM
c1ccccn1ccc(=o)nc2cccc(c23)c(=o)c4c(c3=o)cccc4nc(=o)ccn5ccccc5	**CHEMBL144848 [3]** IC_{50} = 7,8 μM
cn(c)ccc(=o)nc1cccc(c12)c(=o)c3c(c2=o)c(ccc3)nc(=o)ccn(c)c	**CHEMBL144984 [8]** IC_{50} = 6,4 μM

Table S2. *Cont.*

**SMILES chemical formula**	**Reference code and IC_{50}**
cc[n+](c)(cc)ccc(=o)nc1cccc(c12)c(=o)c3c(c2=o)c(ccc3)nc(=o)cc[n+](c)(cc)cc	**CHEMBL145311 [8]** IC_{50} = 7,5 μM
ccn(cc)ccc(=o)nc1cccc(c12)c(=o)c3c(c2=o)c(ccc3)nc(=o)ccn(cc)cc	**CHEMBL145575 [8]** IC_{50} = 4,2 μM
cc(c1)cc2n(cc)c(cccc3)c3c4[n+](cc)c(c5c1c24)cccc5	**CHEMBL154643 [13]** IC_{50} = 2 μM
c1c(cl)ccc(c12)n(cc2)ccn(c(c34)cccc4)cc3cc(=o)ncccccccccccco[p@@](=o)(o)oc5ccccc5	**CHEMBL155030 [14]** IC_{50} = 6,7 μM
cc(c1)cc2n(c)c(ccc(cl)c3)c3c4[n+](c)c(c5c1c24)ccc(cl)c5	**CHEMBL156480 [13]** IC_{50} = 0,25 μM
cc(c1)cc2n(c)c(cccc3)c3c4[n+](c)c(c5c1c24)cccc5	**CHEMBL156492 [13]** IC_{50} = 0,76 μM
c1ccc2n(c)c(cccc3)c3c4[n+](c)c(c5c1c24)cccc5	**CHEMBL157766 [13]** IC_{50} = 0,38 μM
cc(c1)cc2n(c)c(ccc(f)c3)c3c4[n+](c)c(c5c1c24)ccc(f)c5	**CHEMBL158083 [13]** IC_{50} = 0,33 μM
cc(c1)cc2n(c)c(ccc(c)c3)c3c4[n+](c)c(c5c1c24)ccc(c)c5	**CHEMBL158365 [13]** IC_{50} = 0,25 μM
c1cccn1c(=o)ccccc(=o)nc2ccc(cc2)nc3c(ccc(c4)nc(=o)ccn5cccc5)c4nc(c36)cc(cc6)nc(=o)ccn7cccc7	**CHEMBL181970 [7]** IC_{50} = 0,31 μM
ccn(cc)cccccccnc(=o)ccccc(=o)nc1ccc(cc1)nc2c(ccc(c3)nc(=o)ccn4cccc4)c3nc(c25)cc(cc5)nc(=o)ccn6cccc6	**CHEMBL182871 [7]** IC_{50} = 0,08 μM
ccn(cc)ccnc(=o)ccccc(=o)nc1ccc(cc1)nc2c(ccc(c3)nc(=o)ccn4cccc4)c3nc(c25)cc(cc5)nc(=o)ccn6cccc6	**CHEMBL184587 [7]** IC_{50} = 0,09 μM
cn(c)cccnc(c1c(n2)cccc1)c(c2c34)[nh]c3cccc4	**CHEMBL219728 [15]** IC_{50} = 0,63 μM
c1c(cl)c(o)c(o)c(c12)oc(cc2=o)-c3cc(o)c(o)cc3	**CHEMBL222132 [16]** IC_{50} = 0,82 μM

Table S2. *Cont.*

**SMILES chemical formula**	**Reference code and IC_{50}**
c1cc(o)c(o)c(c12)oc(c(f)c2=o)-c3cc(o)c(o)cc3	**CHEMBL222354 [16]** IC_{50} = 0,6 µM
coc(cc1)c(oc)c(c12)oc(cc2=o)-c3cc(n)c(n)cc3	**CHEMBL222490 [16]** IC_{50} = 7,4 µM
c1cc(o)c(o)c(c12)oc(cc2=o)-c3cc(n)c(n)cc3	**CHEMBL222539 [16]** IC_{50} = 3,6 µM
c1cc(o)c(o)c(c12)oc(cc2=o)-c3cc(o)c(o)cc3	**CHEMBL222541 [16]** IC_{50} = 0,2 µM
coc(cc1)c(oc)c(c12)oc(cc2=o)-c(c3)ccc(c34)[nh]cn4	**CHEMBL222547 [16]** IC_{50} = 2,2 µM
c1cc(o)c(o)c(o)c1c(=o)/c=c/c2cc(o)c(o)cc2	**CHEMBL222557 [16]** IC_{50} = 6 µM
n1c(o)[nh]c(c12)ccc(c2)-c(cc3=o)oc(c34)c(o)c(o)cc4	**CHEMBL222757 [16]** IC_{50} = 1,2 µM
c[n+](c)(c)ccn(c(=o)c1c2c34)c(=o)c2ccc4c5c6c7c(c(=o)n(cc[n+](c)(c)c)c(=o)c7cc5)ccc6c3cc1	**CHEMBL224754 [17]** IC_{50} = 0,03 µM
c1ccccn1ccn(c2)cc3ccc4c5ccc6cn(ccn7ccccc7)cc(cc8)c6c5c8c9ccc2c3c49	**CHEMBL224755 [17]** IC_{50} = 0,4 µM
cn(c)ccn(c(=o)c1c2c34)c(=o)c2ccc4c5c6c7c(c(=o)n(ccn(c)c)c(=o)c7cc5)ccc6c3cc1	**CHEMBL225001 [17]** IC_{50} = 0,16 µM
occnccn(c(=o)c1c2c34)c(=o)c2ccc4c5c6c7c(ccc6c3cc1)c(=o)n(c(=o)c7cc5)ccncco	**CHEMBL225205 [17]** IC_{50} = 0,06 µM
c1cc(o)c(o)cc1-c(c(c#n)c2=o)oc(c23)c(o)c(o)cc3	**CHEMBL225211 [16]** IC_{50} = 0,13 µM
coc(cc1)c(o)c(c12)oc(cc2=o)-c3cc(o)c(o)cc3	**CHEMBL225228 [16]** IC_{50} = 7,8 µM
c[n+](c)(c)ccn(c(=o)c1cc2)c(=o)c3ccc4c(=o)n(c(=o)c2c4c13)cc[n+](c)(c)c	**CHEMBL225507 [17]** IC_{50} = 2,5 µM

Table S2. *Cont.*

**SMILES chemical formula**	**Reference code and IC_{50}**
c1cc(o)c(o)cc1c(=o)/c=c/c2cc(o)c(o)cc2	**CHEMBL225618 [16]** IC_{50} = 1,7 μM
c1cccc(c12)[n+](c)cc(c2)nc(=o)c(n3)ccc4ccc(c5c34)ccc(n5)c(=o)nc(c6)c[n+](c)c(c67)cccc7	**CHEMBL259739 [18]** IC_{50} = 0,001 μM
o1cccc1-c2cc(c[n+](c)c2)-c3c(n4)ccc4c(-c(c[n+](c)c5)cc5-c6ccco6)c([nh]7)ccc7c(-c(c[n+](c)c8)cc8-c9ccco9)c([nh]1)ccc1c(c(n1)ccc13)-c(c[n+](c)c1)cc1-c1ccco1	**CHEMBL265594 [19]** IC_{50} = 8 μM
cn(c)cccnc1nc(cccc2)c2c3[nh]c(c4c13)c5c(cc4)cc(cc5)oc	**CHEMBL274096 [2]** IC_{50} = 1 μM
cn(c)cccnc1[nh]c(cccc2)c2c3nc(c4c13)c5c(cc4)ccc(c5)[n+]([o-])=o	**CHEMBL274335 [2]** IC_{50} = 0,5 μM
c[n+](c)(c)cccn(c(=o)c1cc2)c(=o)c3ccc4c(=o)n(c(=o)c2c4c13)ccc[n+](c)(c)c	**CHEMBL275860 [17]** IC_{50} = 2,5 μM
cn(c)cccnc1nc(cccc2)c2c3[nh]c(c4c13)c5c(cc4)ccc(c5)oc	**CHEMBL276202 [2]** IC_{50} = 1,1 μM
c1cccc(c12)ccc(c2)c(\c)=c/c(=o)nc(c3c(=o)o)cccc3	**CHEMBL282105 [4]** IC_{50} = 5 μM
c1c(n)ccc2c1[n+](cc)c(c(c23)cc(n)cc3)-c4ccccc4	**CHEMBL284328 [11]** IC_{50} = 3,3 μM
cn(c)cccn(c(=o)c1cc2)c(=o)c3ccc4c(=o)n(c(=o)c2c4c13)cccn(c)c	**CHEMBL287201 [4]** IC_{50} = 3,5 μM
ccn(cc)ccoc(cc1)cc(c12)o[c@@h]3[c@h]2c(=o)c4c(c3=o)cccc4	**CHEMBL292475 [20]** IC_{50} = 7 μM
cc1ccn(cc1)ccc(=o)nc(cc2)cc(c23)nc4c(c3)ccc(c4)nc(=o)ccn(cc5)ccc5c	**CHEMBL309919 [8]** IC_{50} = 1,35 μM
ccn(cc)ccc(=o)nc(cc1)cc(c12)nc3c(c2)ccc(c3)nc(=o)ccn(cc)cc	**CHEMBL309982 [7]** IC_{50} = 5,8 μM
cn(c)ccc(=o)nc(cc1)cc(c12)nc3c(c2)ccc(c3)nc(=o)ccn(c)c	**CHEMBL311072 [7]** IC_{50} = 8,2 μM

Table S2. *Cont.*

SMILES chemical formula	Reference code and IC_{50}
c1ccccc1c[c@@h](nc(=o)oc(c)(c)c)c(=o)n[c@@h](cc2c[nh]c(c23)cccc3)c(=o)ncccccccccccco[p@@](=o)(o)oc4c(cl)cccc4	**CHEMBL314040 [9]** IC_{50} = 6,8 μM
c1ccccc1coc(=o)n[c@h](cc2c[nh]c(c23)cccc3)c(=o)ncccccccccccco[p@](=o)(o)oc4c(cl)cccc4	**CHEMBL314864 [9]** IC_{50} = 3,6 μM
c1ccc[c@h](co)[n@@+]1(c)ccc(=o)nc2ccc(c(c23)c(=o)c4c(c3=o)cccc4)nc(=o)cc[n@@+]5(c)[c@@h](co)cccc5	**CHEMBL322686 [5]** IC_{50} = 9,4 μM
oc[c@@h]1ccccn1ccc(=o)nc(cc2)cc(c2-3)c(=o)c4c3ccc(c4)nc(=o)ccn5cccc[c@h]5co	**CHEMBL328065 [10]** IC_{50} = 8 μM
c1cccn1ccc(=o)nc(cc2)cc(c23)nc4c(c3ncccn(c)c)ccc(c4)nc(=o)ccn5cccc5	**CHEMBL335132 [9]** IC_{50} = 0,06 μM
c1cccn1ccc(=o)nc(cc2)cc(c23)nc4c(c3nccoc)ccc(c4)nc(=o)ccn5cccc5	**CHEMBL335564 [7]** IC_{50} = 0,14 μM
c1cccc(c12)[n+](c)cc(c2)-c3c(n4)ccc4c(-c(c5)c[n+](c)c(c56)cccc6)c([nh]7)ccc7c(-c8ccc[n+](c)c8)c([nh]9)ccc9c(c(n1)ccc13)-c(c1)c[n+](c)c(c12)cccc2	**CHEMBL335814 [19]** IC_{50} = 5 μM
c1cccn1ccc(=o)nc(cc2)cc(c23)nc4c(c3nc5ccc(n)cc5)ccc(c4)nc(=o)ccn6cccc6	**CHEMBL335819 [7]** IC_{50} = 0,07 μM
c1cccn1ccc(=o)nc(cc2)cc(c23)nc4c(c3nc5cc(n)ccc5)ccc(c4)nc(=o)ccn6cccc6	**CHEMBL336417 [7]** IC_{50} = 0,06 μM
c1cccn1ccc(=o)nc(cc2)cc(c23)nc4c(c3nc(cc5)ccc5n(c)c)ccc(c4)nc(=o)ccn6cccc6	**CHEMBL336434 [17]** IC_{50} = 0,04 μM
c1cccn1ccc(=o)nc(cc2)cc(c23)nc4c(c3nc(cc5)ccc5c(=o)c)ccc(c4)nc(=o)ccn6cccc6	**CHEMBL336444 [7]** IC_{50} = 0,04 μM
c1c[n+](c)ccc1-c2c(n3)ccc3c(-c4cc[n+](c)cc4)c([nh]5)ccc5c(-c6cc[n+](c)cc6)c([nh]7)ccc7c(c(n8)ccc28)-c9cc[n+](c)cc9	**CHEMBL337559 [19]** IC_{50} = 8 μM
c1cccn1ccc(=o)nc(c2)ccc(c23)nc4c(c3nc5ccc(n)cc5)ccc(c4)nc(=o)ccn6cccc6	**CHEMBL337762 [7]** IC_{50} = 0,08 μM
c1cccn1ccc(=o)nc(c2)ccc(c23)nc4c(c3nc(cc5)ccc5n(c)c)cc(cc4)nc(=o)ccn6cccc6	**CHEMBL341985 [7]** IC_{50} = 0,5 μM

Table S2. *Cont.*

**SMILES chemical formula**	**Reference code and IC_{50}**
c1cccn1ccc(=o)nc(c2)ccc(c23)nc4c(c3nc5cc(n)ccc5)ccc(c4)nc(=o)ccn6cccc6	**CHEMBL342480 [7]** IC_{50} = 0,21 μM
c1cccn1ccc(=o)nc(c2)ccc(c23)nc4c(c3nc5cc(oc)ccc5)cc(cc4)nc(=o)ccn6cccc6	**CHEMBL342921 [7]** IC_{50} = 2,73 μM
c1cccn1ccc(=o)nc(c2)ccc(c23)nc4c(c3nc5c(o)cccc5)cc(cc4)nc(=o)ccn6cccc6	**CHEMBL343041 [7]** IC_{50} = 1,03 μM
c1cccc[n+]1(c)ccc(=o)nc2cccc(c23)c(=o)c4c(c3=o)c(ccc4)nc(=o)cc[n+]5(c)ccccc5	**CHEMBL343238 [3]** IC_{50} = 7,8 μM
c1cccn1ccc(=o)nc(c2)ccc(c23)c(=o)c4c(c3=o)ccc(c4)nc(=o)ccn5cccc5	**CHEMBL343445 [8]** IC_{50} = 1,8 μM
c1cccn1ccc(=o)nc(c2)ccc(c23)nc4c(c3nc5c(n)cccc5)ccc(c4)nc(=o)ccn6cccc6	**CHEMBL343609 [7]** IC_{50} = 0,11 μM
c1cccn1ccc(=o)nc(cc2)cc(c23)nc4c(c3nc5ccc(f)cc5)ccc(c4)nc(=o)ccn6cccc6	**CHEMBL343795 [7]** IC_{50} = 0,07 μM
c1ccc[n+]1(c)ccc(=o)nc2cccc(c23)c(=o)c4c(c3=o)cccc4nc(=o)cc[n+]5(c)cccc5	**CHEMBL344072 [8]** IC_{50} = 8,8 μM
c1cccn1ccc(=o)nc(c2)ccc(c23)nc4c(c3nc5c(n)cccc5)cc(cc4)nc(=o)ccn6cccc6	**CHEMBL345035 [8]** IC_{50} = 0,17 μM
c1c(cl)ccc(c12)n(cc2)ccn(c(c34)cccc4)cc3cc(=o)ncccccccccccco[p@@](=o)(o)oc5ccccc5	**CHEMBL345715 [14]** IC_{50} = 8,7 μM
c1cccn1cccn(c(c23)cccc3)cc2cc(=o)nccccccccccco[p@@](=o)(o)c4c(cl)cccc4	**CHEMBL349085 [14]** IC_{50} = 4,2 μM
c1c(cl)ccc(c12)n(cc2)ccn(c(c34)cccc4)cc3cc(=o)ncccccccccccco[p@@](=o)(o)oc5cc(cl)ccc5	**CHEMBL351995 [14]** IC_{50} = 2,5 μM
cn(c)ccc(=o)nc1cccc(c12)c(=o)c3c(c2=o)cccc3nc(=o)ccn(c)c	**CHEMBL356077 [8]** IC_{50} = 1,3 μM
c1ccccn1ccn(c(=o)c2c3c45)c(=o)c3ccc5c6c7c8c(ccc7c4cc2)c(=o)n(c(=o)c8cc6)ccn9ccccc9	**CHEMBL359594 [17]** IC_{50} = 0,2 μM

Table S2. *Cont.*

**SMILES chemical formula**	**Reference code and IC_{50}**
cn(c)cccc(n1)[nh]c(c12)ccc(c2)-c(cc3=o)oc(c34)c(oc)c(cc4)oc	**CHEMBL376279 [16]** IC_{50} = 0,47 µM
n1c[nh]c(c12)ccc(c2)-c(cc3=o)oc(c34)c(o)c(o)cc4	**CHEMBL390725 [16]** IC_{50} = 4 µM
c1cccc(c12)[n+](c)cc(c2)nc(=o)c3cccc(n3)c(=o)nc(c4)c[n+](c)c(c45)cccc5	**CHEMBL409247 [17]** IC_{50} = 0,06 µM
c1cccc(c12)n(cc2c)cccn(c(c34)cccc4)cc3cc(=o)ncccccccccccco[p@](=o)(o)oc5cc(cl)ccc5	**CHEMBL413033 [14]** IC_{50} = 3,4 µM
c1cc(o)c(o)c(c12)oc(cc2=o)-c3ccc(o)cc3	**CHEMBL420982 [16]** IC_{50} = 3 µM
clc1cccc(cl)c1csc(c2c#n)nc(cc2)/c=n/c(c3c)cccn3	**CHEMBL421825 [21]** IC_{50} = 1 µM
c[n+](c)(c)ccc(=o)nc1cccc(c12)c(=o)c3c(c2=o)c(ccc3)nc(=o)cc[n+](c)(c)c	**CHEMBL422120 [3]** IC_{50} = 4,4 µM
c1cc(o)c(o)c(c12)oc(c(cl)c2=o)-c3cc(o)c(o)cc3	**CHEMBL426182 [16]** IC_{50} = 0,8 µM
c[c@h]1ccccn1ccc(=o)nc(cc2)cc(c23)nc4c(c3)ccc(c4)nc(=o)ccn5cccc[c@@h]5c	**CHEMBL431404 [7]** IC_{50} = 3,1 µM
c1cccc(c12)[nh]c(c2)c[c@h](nc(=o)oc(c)(c)c)c(=o)n[c@@h](cc3c[nh]c(c34)cccc4)c(=o)ncccccccccccco[p@](=o)(o)oc5c(cl)cccc5	**CHEMBL432562 [9]** IC_{50} = 0,3 µM
cc1oc(n2)-c(c(o3)c)nc3[c@h](cs4)n=c4c(co5)nc5-c(co6)nc6-c(co7)nc7-c(co8)nc8-c(co9)nc9-c12	**CHEMBL443683 [18]** IC_{50} = 1,15 µM
c1cccn1ccc(=o)nc(cc2)cc(c23)nc4c(ccc(c4)nc(=o)ccn5cccc5)c3ncnccncnc6c(ccc(c7)nc(=o)ccn8cccc8)c7nc(c69)cc(cc9)nc(=o)ccn1cccc1	**CHEMBL443702 [22]** IC_{50} = 7,5 µM
cn(c)ccc(=o)nc(c1)ccc(c12)c(=o)c3c(c2=o)ccc(c3)nc(=o)ccn(c)c	**CHEMBL444347 [8]** IC_{50} = 4,1 µM
c1cccn1ccc(=o)nc(cc2)cc(c23)nc4c(ccc(c4)nc(=o)ccn5cccc5)c3nccccccnc6c(ccc(c7)nc(=o)ccn8cccc8)c7nc(c69)cc(cc9)nc(=o)ccn1cccc1	**CHEMBL448813 [22]** IC_{50} = 4 µM

Table S2. *Cont.*

**SMILES chemical formula**	**Reference code and IC_{50}**
cn(c)cccnc(c1c([n+]2c)cccc1)c(c2c34)oc3cccc4	**CHEMBL482414 [25]** IC_{50} = 0,37 μM
ccn(cc)cccnc(c1c([n+]2c)cccc1)c(c2c34)[nh]c3cccc4	**CHEMBL484670 [25]** IC_{50} = 0,16 μM
cn(c)cccnc(c1c([n+]2c)cccc1)c(c2c34)[nh]c3c(f)cc(f)c4	**CHEMBL489811 [25]** IC_{50} = 0,4 μM
ccn(cc)cccnc(c1c([n+]2c)cccc1)c(c2c34)[nh]c3c(f)cc(f)c4	**CHEMBL490013 [25]** IC_{50} = 0,27 μM
cn(c)cccnc(c1c([n+]2c)cccc1)c(c2c34)[nh]c3ccc(f)c4	**CHEMBL491432 [25]** IC_{50} = 0,31 μM
ccn(cc)cccnc(c1c([n+]2c)cccc1)c(c2c34)[nh]c3ccc(f)c4	**CHEMBL491633 [25]** IC_{50} = 0,2 μM
c1cccn1ccc(=o)nc(cc2)cc(c23)nc4c(ccc(c4)nc(=o)ccn5cccc5)c3ncoccocnc6c(ccc(c7)nc(=o)ccn8cccc8)c7nc(c69)cc(cc9)nc(=o)ccn1cccc1	**CHEMBL506865 [22]** IC_{50} = 9 μM
cn(c)cccnc(c1c([n+]2c)cccc1)c(c2c34)[nh]c3cccc4	**CHEMBL507633 [25]** IC_{50} = 0,22 μM
c1ccc[n+]1(c)ccc(=o)ncc(ccc2)c(c23)nc4c(c3)cccc4cnc(=o)cc[n+]5(c)cccc5	**CHEMBL566078 [1]** IC_{50} = 4 μM
c[n+](c)(c)cc(=o)ncc(ccc1)c(c12)nc3c(c2)cccc3cnc(=o)c[n+](c)(c)c	**CHEMBL572616 [1]** IC_{50} = 7,3 μM
cn(c)ccncc1cccc(c12)cc3c(n2)c(ccc3)cnccn(c)c	**CHEMBL574042 [1]** IC_{50} = 5,6 μM
c[n+](c)(c)ccc(=o)ncc(ccc1)c(c12)nc3c(c2)cccc3cnc(=o)cc[n+](c)(c)c	**CHEMBL575809 [1]** IC_{50} = 3 μM
c1cccn1ccc(=o)ncc(ccc2)c(c23)nc4c(c3)cccc4cnc(=o)ccn5cccc5	**CHEMBL5771$_{50}$[1]** IC_{50} = 6 μM
c1ccccn1ccc(=o)nc(c2)ccc(c23)c(=o)[c@@h]4[c@@h](c3=o)c=cc(=c4)nc(=o)ccn5ccccc5	**CHEMBL609670 [6]** IC_{50} = 4,5 μM

c1cccn1ccc(=o)nc(c2)ccc(c23)c(=o)[c@h]4[c@h](c3=o)c=cc(=c4)nc(=o)ccn5cccc5	**CHEMBL612102 [6]** IC_{50} = 1,8 μM
c[c@h]1ccccn1ccc(=o)nc(c2)ccc(c23)n=c4[c@@h](c3=o)c=c(c=c4)nc(=o)ccn5cccc[c@@h]5c	**CHEMBL1076521 [7]** IC_{50} = 1,5 μM
occ[c@h]1ccccn1ccc(=o)nc(cc2)cc(c23)n=c4[c@h](c3=o)c=c(c=c4)nc(=o)ccn5cccc[c@h]5cco	**CHEMBL1077669 [7]** IC_{50} = 0,2 μM
ccn(cc)ccc(=o)nc(cc1)cc(c12)n=c3[c@h](c2=o)c=c(c=c3)nc(=o)ccn(cc)cc	**CHEMBL1077950 [7]** IC_{50} = 0,7 μM
c1cccc[n+]1([o-])c(=o)ccccc(=o)nc2ccc(cc2)nc3c(ccc(c4)nc(=o)ccn5cccc5)c4nc(c36)cc(cc6)nc(=o)ccn7cccc7	**CHEMBL1078222 [7]** IC_{50} = 0,27 μM
c1cccn1ccc(=o)nc(cc2)cc(c23)nc4c(c3nc5cccccc5)ccc(c4)nc(=o)ccn6cccc6	**CHEMBL1078502 [7]** IC_{50} = 0,21 μM
c1cccn1ccc(=o)nc(c2)ccc(c23)n=c4[c@@h](c3=o)c=c(c=c4)nc(=o)ccn5cccc5	**CHEMBL1078516 [7]** IC_{50} = 5,8 μM
oc1ccn(cc1)ccc(=o)nc(c2)ccc(c23)n=c4[c@h](c3=o)c=c(c=c4)nc(=o)ccn(cc5)ccc5o	**CHEMBL1078525 [7]** IC_{50} = 2,3 μM
cn(c)ccc(=o)nc(c1)ccc(c12)n=c3[c@h](c2=o)c=c(c=c3)nc(=o)ccn(c)c	**CHEMBL1078925 [7]** IC_{50} = 0,6 μM
c1cncc[n+]1(c)ccc(=o)nc(c2)ccc(c23)n=c4[c@h](c3=o)c=c(c=c4)nc(=o)cc[n+]5(c)ccncc5	**CHEMBL1079036 [7]** IC_{50} = 1,9 μM
c1ccccn1ccc(=o)nc(c2)ccc(c23)n=c4[c@@h](c3=o)c=c(c=c4)nc(=o)ccn5ccccc5	**CHEMBL1079201 [7]** IC_{50} = 1,9 μM
cc1ccn(cc1)ccc(=o)nc(cc2)cc(c23)n=c4[c@@h](c3=o)c=cc(=c4)nc(=o)ccn(cc5)ccc5c	**CHEMBL1080613 [7]** IC_{50} = 1,7 μM
c1nc(cl)ccc1coc(cc2o)cc(c23)oc(cc3=o)-c4ccc(cc4)occ5ccc(cl)nc5	**CHEMBL1169924 [23]** IC_{50} = 0,8 μM
c1nc(cl)ccc1coc(cc2)cc(c23)occ(c3=o)-c4ccc(cc4)oc	**CHEMBL1170488 [13]** IC_{50} = 2,5 μM
c1nc(cl)ccc1coc(cc2)cc(c23)oc(cc3=o)-c4ccc(cc4)oc	**CHEMBL1170702 [23]** IC_{50} = 3,1 μM

Table S2. *Cont.*

SMILES chemical formula	Reference code and IC_{50}
c1nc(cl)ccc1coc(cc2)ccc2-c(cc3=o)oc(c34)cc(o)cc4o	**CHEMBL1172458 [23]** IC_{50} = 3,5 μM
c1cc(o)ccc1[c@@h](c2)n(c(=o)c)n=c2c(c3)c(=o)oc(c34)cccc4br	**CHEMBL1257134 [23]** IC_{50} = 4 μM
c1cccc(cl)c1[c@h](c2)n(c(=o)c)n=c2c(c3)c(=o)oc(c34)cccc4	**CHEMBL1258868 [23]** IC_{50} = 6,5 μM
c1nc(cl)ccc1csc(o2)nnc2-c3cccc(c34)cccc4	**CHEMBL1276872 [23]** IC_{50} = 4,2 μM
c1nc(cl)ccc1csc(o2)nnc2-c3c(o)cc(cc3)oc	**CHEMBL1278128 [23]** IC_{50} = 2,3 μM
cn(c)cccn1ccn(cc1)c(c2)c3c4ccc(c(=o)n(c(=o)c5cc6)ccn7ccccc7)c5c4c6c8ccc9c(=o)n(c(=o)c2c9c38)ccn1ccccc1	**CHEMBL1689442 [24]** IC_{50} = 3 μM
c1ccccc1c(=o)n(n=c2c)[c@h](c2)c3c(o)cccc3	**CHEMBL1770486 [24]** IC_{50} = 4 μM
c1ccc(c)cc1csc(o2)nnc2-c(c3)ccc(c34)occo4	**CHEMBL1917259 [24]** IC_{50} = 3,26 μM
s1cc(c)[n+](cc)c1/c=c2/n(cc)c(=o)/c(s2)=c(/n3c)sc(c34)cccc4	**CHEMBL1956189 [25]** IC_{50} = 2 μM
oc1ccn(cc1)cc(=o)n(n=c2c)[c@h](c2)c3c(o)cccc3	**CHEMBL2012469 [25]** IC_{50} = 9,11 μM
fc(f)(f)c1ccn(cc1)cc(=o)n(n=c2c)[c@h](c2)c3c(o)cccc3	**CHEMBL2012474 [25]** IC_{50} = 8,81 μM
cc1ccn(cc1)cc(=o)n([c@h](c2)c3ccccc3)n=c2c(c4)c(=o)oc(c45)cccc5	**CHEMBL2012477 [25]** IC_{50} = 2 μM

References

1. Zhang, X.; Qiu, M.; Sun, J.; Zhang, Y.; Yang, Y.; Wang, X.; Tang, J.; Zhu, J. Synthesis, biological evaluation, and molecular docking studies of 1,3,4-oxadiazole derivatives possessing 1,4-benzodioxan moiety as potential anticancer agents. *Bioorg. Med. Chem.* **2011**, *19*, 6518–6524.
2. Alberti, P.; Schmitt, P.; Nguyen, C.; Rivalle, C.; Hoarau, M.; Grierson, D.S.; Mergnya J.L. Benzoindoloquinolines Interact with DNA Tetraplexes and Inhibit Telomerase. *Bioorg. Med. Chem. Lett.* **2002**, *12*, 1071–1074.
3. Perry, P.J.; Reszka, A.P.; Wood, A.A.; Read, M.A.; Gowan, S.M.; Dosanjh, H.S.; Trent, J.O.; Jenkins, T.C.; Kelland, L.R.; Neidle, S. Human Telomerase Inhibition by Regioisomeric Disubstituted Amidoanthracene-9,10-diones. *J. Med. Chem.* **1998**, *41*, 4873–4884.
4. Barma, D.K.; Elayadi, A.; Falck, J.R.; Corey, D.R. Inhibition of Telomerase by BIBR 1532 and Related Analogues. *Bioorg. Med. Chem. Lett.* **2003**, *13*, 1333–1336.
5. Perry, P.J.; Gowan, S.M.; Reszka, A.P.; Polucci, P.; Jenkins, T.C.; Kelland, L.R.; Neidle, S. 1,4- and 2,6-Disubstituted amidoanthracene-9,10-dione derivatives as inhibitors of human telomerase. *J. Med. Chem.* **1998**, *41*, 3253–3260.
6. Read, M.A.; Wood, A.A.; Harrison, J.R.; Gowan, S.M.; Kelland, L.R.; Dosanjh, H.S.; Neidle, S. Molecular modeling studies on G-quadruplex complexes of telomerase inhibitors: Structure-activity relationships. *J. Med. Chem.* **1999**, *42*, 4538–4546.
7. Castillo-González, D.; Cabrera-Pérez, M.A.; Pérez-González, M.; Morales Helguera, A.; Durán-Martínez, A. Prediction of telomerase inhibitory activity for acridinic derivatives based on chemical structure. *Eur. J. Med. Chem.* **2009**, *44*, 4826–4840.
8. Harrison, R.J.; Gowan, S.M.; Kelland, L.R.; Neidle, S. Human telomerase inhibition by substituted acridine derivatives. *Bioorg. Med. Chem. Lett.* **1999**, *9*, 2463–2468.
9. Sasaki, S.; Ehara, T.; Alam, M.R.; Fujino, Y.; Harada, N.; Kimura, J.; Nakamura, H.; Maeda, M. Solid-phase synthesis of a library constructed of aromatic phosphate, long alkyl chains and tryptophane components, and identification of potent dipeptide telomerase inhibitors. *Bioorg. Med. Chem. Lett.* **2001**, *11*, 2581–2584.
10. Perry, P.J.; Read, M.A.; Davies, R.T.; Gowan, S.M.; Reszka, A.P.; Wood, A.A.; Kelland, L.R.; Neidle, S. 2,7-disubstituted amidofluorenone derivatives as inhibitors of human telomerase. *J. Med. Chem.* **1999**, *42*, 2679–2684.
11. West, C.; Francis, R.; Friedman, S.H. Small molecule/nucleic acid affinity chromatography: Application for the identification of telomerase inhibitors which target its key RNA/DNA heteroduplex. *Bioorg. Med. Chem. Lett*. **2001**, *11*, 2727–2730.
12. Huang, H.S.; Huang, K.F.; Li, C.L.; Huang, Y.Y.; Chiang, Y.H.; Huang, F.C.; Lin, J.J. Synthesis, human telomerase inhibition and anti-proliferative studies of a series of 2,7 bis-substituted amido-anthraquinone derivatives. *Bioorg. Med. Chem.* **2008**, *16*, 6976–6986.
13. Heald, R.A.; Modi, C.; Cookson, J.C.; Hutchinson, I.; Laughton, C.A.; Gowan, S.M.; Kelland, L.R.; Stevens, M.F. Antitumor polycyclic acridines. 8.1 synthesis and telomerase-inhibitory activity of methylated pentacyclic acridinium salts. *J. Med. Chem.* **2002**, *45*, 590–597.

14. Sasaki, S.; Ehara, T.; Sakata, I.; Fujino, Y.; Harada, N.; Kimura, J.; Nakamura, H.; Maeda, M. Development of novel telomerase inhibitors based on a bisindole unit. *Bioorg. Med. Chem. Lett.* **2001**, *11*, 583–585.
15. Lu, Y.J.; Ou, T.M.; Tan, J.H.; Hou, J.Q.; Shao, W.Y.; Peng, D.; Sun, N.; Wang, X.D.; Wu, W.B.; Bu, X.Z.; *et al.* 5-*N*-methylated quindoline derivatives as telomeric G-quadruplex stabilizing ligands: Effects of 5-*N* positive charge on quadruplex binding affinity and cell proliferation. *J. Med. Chem.* **2008**, *51*, 6381–6392.
16. Menichincheri, M.; Ballinari, D.; Bargiotti, A.; Bonomini, L.; Ceccarelli, W.; D'Alessio, R.; Fretta, A.; Moll, J.; Polucci, P.; Soncini, C.; *et al.* Catecholic flavonoids acting as telomerase inhibitors. *J. Med. Chem.* **2004**, *47*, 6466–6475.
17. Sissi, C.; Lucatello, L.; Krapcho, A.P.; Maloney, D.J.; Boxer, M.B.; Camarasa, M.V.; Pezzoni, G.; Mentac, E.; Palumbo, M. Tri-, tetra- and heptacyclic perylene analogues as new potential antineoplastic agents based on DNA telomerase inhibition. *Bioorg. Med. Chem.* **2007**, *15*, 555–562.
18. De Cian, A.; Cristofari, G.; Reichenbach, P.; de Lemos, E.; Monchaud, P.; Teulade-Fichou, M.P.; Shin-ya, K.; Lacroix, L.; Lingner, J.; Mergny, J.L. Reevaluation of telomerase inhibition by quadruplex ligands and their mechanisms of action. *Proc. Natl. Acad. Sci. USA* **2007**, *104*, 17347–17352.
19. Shi, D.F.; Wheelhouse, R.T.; Sun, D.; Hurley, L.H. Quadruplex-interactive agents as telomerase inhibitors: Synthesis of porphyrins and structure-activity relationship for the inhibition of telomerase. *J. Med. Chem.* **2001**, *44*, 4509–4523.
20. Caprio, V.; Guyen, B.; Opoku-Boahen, Y.; Mann, J.; Gowan, S.M.; Kelland, L.M.; Readd, M.A.; Neidle, S. A novel inhibitor of human telomerase derived from 10H-Indolo[3,2-b]quinoline. *Bioorg. Med. Chem. Lett.* **2000**, *10*, 2063–2066.
21. Jew, S.S.; Park, B.S.; Lim, D.Y.; Kim, M.G.; Chung, I.K.; Kim, J.H.; Hong, C.I.; Kim, J.K.; Park, H.J.; Lee, J.H.; *et al.* Synthesis of 6-formyl-pyridine-2-carboxylate derivatives and their telomerase inhibitory activities. *Bioorg. Med. Chem. Lett.* **2003**, *13*, 609–612.
22. Fu, Y.T.; Keppler, B.R.; Soares, J.; Jarstfer, M.B. BRACO19 analog dimers with improved inhibition of telomerase and hPot 1. *Bioorg. Med. Chem.* **2009**, *17*, 2030–2037.
23. Liu, X.H.; Liu, H.F.; Shen, X.; Song, B.A.; Bhadury, P.S.; Zhu, H.L.; Liu, J.X.; Qi, X.B. Synthesis and molecular docking studies of novel 2-chloro-pyridine derivatives containing flavone moieties as potential antitumor agents. *Bioorg. Med. Chem. Lett.* **2010**, *20*, 4163–4167.
24. Casagrande, V.; Salvati, E.; Alvino, A.; Bianco, A.D.; Ciammaichella, A.; D'Angelo, C.; Ginnari-Satriani, L.; Serrilli, A.M.; Iachettini, S.; Leonetti, C.; *et al.* N-cyclic bay-substituted perylene G-quadruplex ligands have selective antiproliferative effects on cancer cells and induce telomere damage. *J. Med. Chem.* **2011**, *54*, 1140–1156.
25. Kong, D.; Yamori, T. JFCR39, a panel of 39 human cancer cell lines, and its application in the discovery and development of anticancer drugs. *Bioorg. Med. Chem.* **2012**, *20*, 1947–1951.

© 2013 by the authors; licensee MDPI, Basel, Switzerland. This article is an open access article distributed under the terms and conditions of the Creative Commons Attribution license (http://creativecommons.org/licenses/by/3.0/).
